# National health research system in Malawi: dead, moribund, tepid or flourishing?

**DOI:** 10.1186/s12913-015-0796-1

**Published:** 2015-03-31

**Authors:** Joses Muthuri Kirigia, Damson D Kathyola, Adamson S Muula, Martin Matthew Okechukwu Ota

**Affiliations:** Research, Publications and Library Services Programme, Health Systems and Services Cluster, World Health Organization, Regional Office for Africa, Brazzaville, Congo; Ministry of Health, Department of Research, Lilongwe, Malawi; Department of Community Health, Faculty of Public Health and Family Medicine, College of Medicine, Blantyre, Malawi

**Keywords:** National health research system, Stewardship, Governance, Developing and sustaining resources, Financing, Producing and using research

## Abstract

**Background:**

Several instruments at both the global and regional levels to which countries in the WHO African Region are party call for action by governments to strengthen national health research systems (NHRS). This paper debates the extent to which Malawi has fulfilled this commitment.

**Discussion:**

Some research literature has characterized African research – and by implication NHRS – as moribund. In our view, the Malawi government, with partner support, has made effort to strengthen the capacities of individuals and institutions that generate scientific knowledge. This is reflected in the Malawi national NHRS index (MNSR4HI) of 51%, which is within the 50%-69% range, and thus, it should be characterized as tepid with significant potential to flourish. Governance of research for health (R4H) has improved with the promulgation of the Malawi Science and Technology Act in 2003. However, lack of an explicit R4H policy, a strategic plan and a national R4H management forum undermines the government’s effectiveness in overseeing the operation of the NHRS. The mean index of ‘governance of R4H’ sub-functions was 67%, implying that research governance is tepid. Malawi has a national health research focal point, an R4H program, and four public and 11 private universities. The average index of ‘creating and sustaining resources’ sub-functions was 48.6%, meaning that R4H human and infrastructural resources can be considered to be in a moribund state. The average index of ‘producing and using research’ sub-functions of 50.4% implies that production and utilization of research findings in policy development and public health practice can best be described as tepid. Efforts need to be intensified to boost national research productivity. Over the five financial years 2011–2016 the government plans to spend 0.26% of its total health budget on R4H. The mean index of ‘financing’ sub-functions of 23.6% is within the range of 1-49%, which is considered moribund.

**Summary:**

A functional NHRS is a prerequisite for the achievement of the health system goal of universal health coverage. Malawi, like majority of African countries, needs to invest more in strengthening R4H governance, developing and sustaining R4H resources, and producing and using research findings.

## Background

Malawi is situated in southern Africa and had an estimated population of 15.4 million in 2011 [1]. It is a low income country and in 2011 had a gross national per capita income of about Int$ 870 [1]. In 2009, 39% of the population lived below the international poverty line of less than one United States dollar per day [2]. In 2010, the country lost 12.51 million disability adjusted life years (189 877 deaths), of which 8.96 million (71.6%) were from communicable, maternal, neonatal and nutritional conditions; 2.7 million (21.4%) from noncommunicable diseases; and 0.88 million (7.0%) from injuries [3]. Some of the disease burden associated with communicable and noncommunicable diseases could be attributed to the risk factors contained in Table 1.

**Table 1:** **Risk factors in Malawi compared to African Region and global averages**

**Health risk factors**		**Malawi**	**African Region**	**Global**
Population using improved drinking-water sources (%) (2011)		84	64	89
Population using improved sanitation (%) (2011)		53	34	64
Population using solid fuels (%) (2010)		>95	77	41
Preterm birth rate (per 100 live births) (2010)		18	12	11
Infants exclusively breastfed for the first 6 months of life (%) (2012)		71	35	38
Children aged < 5 years (%) (2012)	Wasted	4.1	10.4	8.0
	Stunted	47.8	40.9	25.7
	Underweight	13.8	25.2	15.7
	Overweight	9.2	7.9	6.6
Prevalence of raised fasting blood glucose among adults aged ≥ 25 years (%) (2008)	Male	6.4	8.3	9.2
	Female	6.2	9.8	9.2
Prevalence of raised blood pressure among adults aged ≥ 25 years (%) (2008)	Male	44.5	38.1	29.2
	Female	39.4	35.5	24.8
Adults aged ≥20 years who are obese (%) (2008)	Male	2.6	5.3	10.0
	Female	6.2	11.1	14.0
Alcohol consumption among adults aged ≥15 years (litres of pure alcohol per person per year) (2008)		1.4	–	–
Prevalence of smoking any tobacco product among adults aged ≥15 years (%) (2009)	Male	26	17	36
	Female	4	3	8
Prevalence of current tobacco use among adolescents aged 13–15 years (%) (2010)	Male	17	20	18
	Female	11	13	11
Prevalence of condom use by adults aged 15–49 years during higher risk sex (%) (2011)	Male	25	–	–
	Female	27	–	–
Population aged 15–24 years with comprehensive correct knowledge of HIV/AIDS (%) (2011)	Male	–	34	–
	Female	–	28	–

Source: WHO [1].

The Malawi health system’s infrastructure comprises 606 health facilities. Of these, 0.7% are central/tertiary hospitals, 3.8% district hospitals, 0.3% mental hospitals, 6.1% community/rural hospitals, 3.5% other hospital types, 69.8% health centers, 12.7% dispensaries, 2.8% maternity units, and 0.3% rehabilitative units. The Christian Health Association of Malawi (CHAM) owns 26.7% of the facilities, local governments 5.1%, the Ministry of Health 59.6%, the Ministry of Health and CHAM 0.2%, and the Ministry of Health and local government 8.4% [2]. The health sector has 215 physicians, 2505 nursing and midwifery personnel, 16 dentistry personnel, 107 pharmaceutical personnel, and 88 environment and public health workers [1].

In 2011, per capita total expenditure on health in Malawi was US$ 30.93 (Int$ 76.99) [1]. About 73.4% of total health expenditure came from general government allocations while the remaining 26.6% was from private sources, of which 53.4% was household out-of-pocket payments. In 2009, out-of-pocket health expenditure as a proportion of total health expenditure was 10% [4]. This was fairly lower than what is considered the threshold level of 15–20%, where the incidence of financial catastrophe caused by out-of-pocket health expenses is significant [5]. Donor funding channeled through public and private entities made up 52.4% of total expenditure on health [6].

Majority of deaths could have been averted if available cost-effective interventions or health services were accessible to those in need of them. But, owing to health system weaknesses, coverage of vital health services is low (Table 2). Research for health (R4H) is essential in development of solutions to overcome health systems weaknesses and in monitoring achievement of the health systems goals of improving health, social and financial risk protection, and health systems’ responsiveness and efficiency.

**Table 2:** **Health services coverage in Malawi compared to African Region and global averages**

**Health services**		**Malawi**	**African Region**	**Global**
Unmet need for family planning (%) (2012)		26	25	12
Contraceptive prevalence (%) 2012)		46	27	63
Antenatal care coverage: at least 4 visits (%) (2012)		46	43	55
Births attended by skilled health personnel (%)		71	49	70
Births by caesarean section (%)		5	4	16
Postnatal care visit within two days of childbirth (%) (2011)		43	45	49
Neonates protected at birth against neonatal tetanus (%) (2011)		87	77	82
Immunization coverage among 1-year-olds (%) (2011)	Measles	96	75	84
	DTP3	97	71	83
	HepB3	97	71	75
	Hib3	97	61	43
Children aged 6–59 months who received vitamin A supplementation (%) (2011)		86	65	50
Children aged < 5 years (%)	With ARI symptoms taken to a health facility (2011)	70	48	78
	With ARI symptoms receiving antibiotics (2011)	–	24	-
	With diarrhoea receiving ORT (ORS and/or RHF) (2011)	69	42	64
	Sleeping under insecticide treated nets (2011)	39	32	–
	With fever who received treatment with any antimalarial (2012)	43	–	–
Pregnant women with HIV receiving antiretrovirals to prevent MTCT (%) (2011)		53	59	57
Antiretroviral therapy coverage among people with advanced HIV infection (%) (2011)		67	57	54
Case-detection rate for all forms of tuberculosis (%)		66	61	67
Treatment-success rate for smear-positive tuberculosis (%)		87	82	87

Source: WHO [1].

A functioning national health research system (NHRS) is needed to generate scientific knowledge and promote its use in the pursuit of universal health coverage [7]. Such a system could shed light on several pertinent health system issues [8,9] including (a) performance of national and district health systems; (b) leadership and governance of the national health system; (c) design and development of a sustainable health financing system; (d) production, management and retention of health workers; (e) management of medical products, including their planning, procurement, storage, distribution and dispensing; (f) development and evaluation of new health technology; (g) economic efficiency of health facilities; (h) attitudinal, cultural, geographical, communication and socioeconomic barriers to health services access; (i) cost-effective ways of scaling up essential health interventions; (j) equity in distribution of health inputs, services and costs; (k) feasibility of various options for attaining universal health coverage; and (l) multisectoral action to address determinants of health.

Global and regional instruments such as the 2010 Sixty-third World Health Assembly [10], the 2008 Bamako Call for Action [11], the 2008 Algiers Declaration [12], the 2006 Abuja and Accra communiques [13,14], the 2005 Fifty-eighth World Health Assembly [15], the 2004 Mexico Ministerial Summit statement [16], and the 1998 Forty-eighth WHO Regional Committee for Africa [17] urged national governments to build and strengthen NHRS to promote the generation of scientific knowledge and promote its utilization in health policy development, planning and decision making.

In 2011, the Malawi Ministry of Health developed a national health sector strategic plan 2011–2016 with the objective to coordinate and regulate health research in such a way that it generates information that will inform policy (and plan) development and evidence-based decision making in programme implementation [2]. Four strategies are stated in the strategic plan for achieving this research objective: to build capacity for high-quality health research at all levels, to strengthen the governance and stewardship role of the health ministry in the conduct of health research, to mobilize resources for health research, and to promote the utilization of research findings for policy and program formulation [2]. Although not explicitly stated, the first objective also covers developing and sustaining R4H resources, and the last objective includes production of research.

This paper debates the questions: To what extent has Malawi implemented the commitments it made at global and regional forums to improve performance of its NHRS? Is the Malawian NHRS dead, moribund, tepid or flourishing? The specific objective is to debate the extent to which Malawi has strengthened its NHRS.

## Discussion

### NHRS conceptual framework

A NHRS is the people, institutions and activities whose primary purpose is to generate and promote utilization of high-quality scientific knowledge to promote, restore and/or maintain the health status of populations [18].

**Figure 1:**
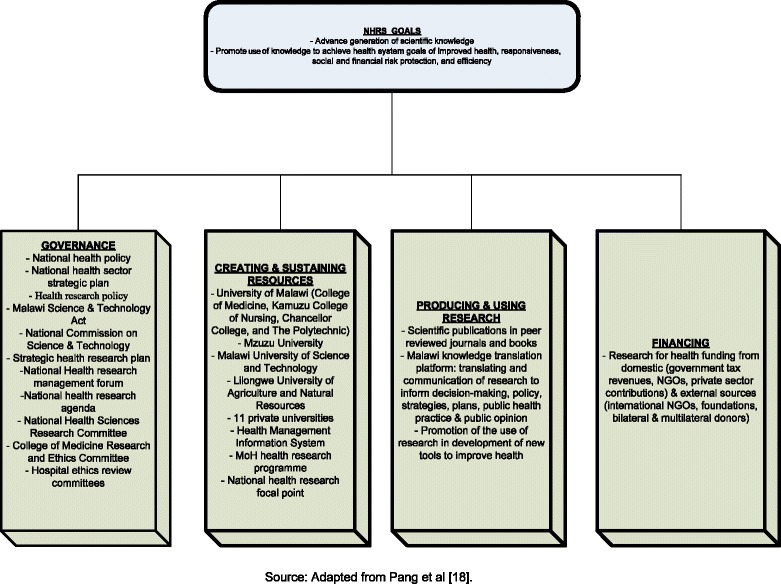
**Malawi national health research systems conceptual framework.**

Figure 1 shows the framework of a NHRS. Its goals are to advance scientific knowledge and promote its utilization in augmenting the performance of the national health system in the achievement of its ultimate objectives of improving health, social and financial risk protection, and health systems’ responsiveness and efficiency. A NHRS has four functions: governance, developing and sustaining resources, financing, and producing and using R4H. Information on the status of the four functions was collected by the Malawi national focal point for R4H (DDK – co-author) using the questionnaire developed and pilot tested by Kirigia and Wambebe [19] and subsequently applied in 2008 by Bondji *et al.* [20]. The questionnaire covered topics relating to health research policy, legislation and strategic planning; research coordination mechanisms; health research programs; research institutes; national universities; research financing and budget; involvement of nongovernmental organizations in research; and actions needed to strengthen health research capacity.

Much of the information on financing for R4H and production and use of research was garnered through review of relevant published and unpublished literature. The information was gathered primarily to underpin the debate and not to generate comprehensive “hard” evidence on the Malawian NHRS.

### Governance of R4H

Government oversight of R4H involves defining the NHRS vision, setting national priorities and overseeing adherence to them, developing and monitoring ethical standards for health research and research partnerships, and monitoring and evaluating the entire NHRS [18]. Specifically, R4H governance concerns development of national health policy and health strategic plan, R4H policy and strategic plan, R4H agenda, R4H legislation, codes of conduct, and ethical standards and guidelines. It includes managing the establishment of a national health research management forum; national, institutional and hospital ethical review committees to protect the dignity, integrity and safety of research participants; national and institutional scientific review committees to ensure scientific rigor of research protocols and their implementation; and the national network for R4H. Designation of a national R4H focal point is required to act as a point of reference for all issues relating to health research. Governance is an overarching function of the government, aiming to ensure effective supervision, coalition building, system design, accountability and regulation for all R4H taking place in both public and private sectors [21]. Table 3 shows the NHRS components in Malawi.

**Table 3:** **NHRS components in Malawi**

**NHRS component**	**Present or not**
National health policy	Yes
Strategic health plan	Yes
Health research policy	No
Law relating to health research	Yes
Law includes ethical concerns	Yes
Strategic health research plan	No
National health research management forum	No
Functional ethical review committee	Yes
Functional scientific review committee	Yes
Institutions with institutional review committees	Yes
Hospitals with ethical review committees	Yes
National health research focal point in the country	Yes
Guidelines for development of collaboration agreements on health research involving health institutions and agencies outside the country	Yes
Health research program	Yes
National health research agenda	Yes
Health research program action plan	Yes
Knowledge translation platform	Yes
Health research program conducts research	No
National health research institute(s) or council	No
Universities with faculties of health sciences that conduct research	Yes
Faculties of health sciences with memorandum of understanding with Ministry of Health	Yes
Budget line for research for health in Ministry of Health budget	Yes
NGOs that undertake health research	Yes

Malawi has a national health policy, a strategic health plan, and a health research agenda but not a valid R4H policy or a strategic R4H plan***.*** The law on health research, the Malawi Science and Technology Act that was promulgated in 2003, encapsulates ethical considerations and other guidelines [22]. The country does not have a functional health research management forum.

The National Commission on Science and Technology (NCST) regulates the conduct of research by the various institutions in Malawi. It has delegated powers to the National Health Sciences Research Committee and the College of Medicine Research and Ethics Committee (COMREC) to review study proposals to ensure methodological and scientific rigor of research protocols and approve health-related research. COMREC and hospital ethical review committees appraise all clinical research proposals to protect the integrity and safety of persons participating in research [22]. Malawi has national guidelines on health research collaborative agreements involving external health institutions and agencies.

What are the implications for Malawi of the absence of a research management forum, which is governance and management mechanism, and a health research policy and a strategic plan, which are the foundations of a NHRS?

Malawi, like any other country, requires an overarching health research management forum with representation of all key stakeholders, including the ministries of education and science and technology, the private sector, health development partners, NGOs, civil society, and the media, and with the Ministry of Health as its Secretariat. Some of its terms of reference could be to (a) advice on national health research policies, strategies and priorities; (b) coordinate R4H; (c) prepare rolling annual national health research plans and monitor and evaluate their implementation; (d) promote the development of health research activities; (e) review R4H management and suggest strategies to overcome problems in implementation of policies; (f) propose mechanisms to nurture a scientific environment to attract talent and develop human resources for R4H; and (g) facilitate dissemination and translation of research results into products, policies and programs aimed at improving health [23,24]. The health research management forum is a vital organ that helps ensure that the government is in the driver’s seat of R4H and that research in the country is harmonized and aligned with the national R4H policy and agenda.

A R4H policy is an essential tool for the government, and when properly formulated in consultation with all relevant stakeholders and implemented through plans and programs, it might have substantial impact on the effectiveness of the NHRS and performance of the national health system. The policy is an official government statement conveying the vision, values, principles, objectives and areas of action to improve the achievement of NHRS’ goals. Its formulation will involve several steps such as (a) gathering information and data for policy development; (b) gathering evidence for effective strategies, including learning from other countries’ experiences; (c) political consultation and negotiation with key stakeholders; (d) definition of the policy vision, values, principles and objectives; (e) translation of objectives into broad areas of action; and (f) identification of the major roles and responsibilities of the various sectors [25]*.* In short, a policy serves to provide a vision that all stakeholders can rally around and strategic direction for R4H that ensures focus on national research priorities.

Health researchers [26,27], policy-makers [10,17,28] and institutions [29,30] consider R4H policy as important for governance and performance of an NHRS. The landmark Mexico City Ministerial Summit on Health Research of November 2004 [15] and the Bamako Ministerial Summit on Research for Health called upon governments to establish and implement a national health research policy [11]. Our literature exploration did not reveal evidence of a correlation between the existence of a R4H policy and research activity or impact on health indicators. However, we hypothesize that the lack of a nationally agreed upon R4H policy might partially explain why, for example the USA, whose healthy life expectancy is 70 years, is nine and eight years lower than those of Japan and Sweden, respectively (see Table 4) [1] in spite that USA’s per capita total expenditure on health is more than double that of those two countries. Even though the USA had the largest number of publications in 2010, Sweden had a higher research output per person in the population [1,31]. Japan, on the other hand, had a lower number of publications per person. So, even though its research output per person is lower than that of the USA, Japan’s policy to guide research contributes to ensuring that majority of the research findings are used in the development of health technologies, and, hence, to improve healthy life expectancy.

**Table 4:** **Comparison of health life expectancy, per capita expenditure on health, and publications per person among 13 countries**

**Countries**	**Healthy life expectancy**	**Per capita total expenditure on health (PPP Int$)**	**Publications per person**
Denmark	70	4456	0.00209
Sweden	78	3938	0.00208
Netherlands	71	5118	0.00183
Finland	71	3382	0.00181
Australia	73	3890	0.00168
Singapore	75	2556	0.00166
Canada	72	4551	0.00154
UK	71	3364	0.00143
Austria	71	4795	0.00133
Germany	71	4474	0.00105
USA	70	8467	0.00104
France	72	4128	0.00097
Japan	79	3415	0.00057

Source: WHO [1] and Pouris [31]. Note: Publications per person equals total number of publications in 2010 divided by total population.

The strategic R4H plan is a document containing national health research strategies, timeframes, indicators and targets, detailed activities by each area of action, estimates of capital and recurrent costs per strategic area, and resources and budget for each year of the plan. The strategic plan is implemented through rolling annual work plans and accompanying annual budgets. Developing the strategic plan involves a number of steps: (a) preparing to develop the plan by gathering the requisite resources, (b) clarifying the mandate and scope of work, (c) analysing external and internal environments, (d) identifying strategic issues, (e) defining the strategic aims and the strategies to address each strategic aim, (f) identifying the resources required to achieve the strategic aims, (g) drawing up an internal capacity building plan, and (h) costing the plan [24,32].

Lack of an explicit R4H policy, a strategic plan or a health research management forum emasculates the government’s effectiveness in supervising the NHRS. The Malawi health sector’s strategic plan acknowledges that the absence of legal and policy frameworks to regulate research and the weak coordination and monitoring of research being carried out in the country pose major challenges to NHRS governance [2]. To address those challenges, the national health sector’s strategic plan has as its research objective to “… coordinate and regulate health research in such a way that it generates information that will inform policy development and evidence-based decision making in programme implementation” ([2] p. 69).

The Ministry of Health plans to strengthen its governance role in R4H through six interventions: (a) implementing the national health research agenda; (b) developing and implementing a national health research policy; (c) supporting the National Health Sciences Research Committee in the review and approval of research proposals; (d) establishing a national public health institute in the Community Health Sciences Unit with leadership over public health research as one of its core functions; (e) ensuring that all health research institutions sign a memorandum of understanding with the health ministry; and (f) supporting regular inspection of and monitoring visits for all health research institutions [2].

### Developing and sustaining R4H resources

Developing and sustaining R4H resources includes building, reinforcing and sustaining of (a) human resources for research in biomedical, bioscience, epidemiology, social science and health systems areas; (b) physical infrastructure; and (c) institutional and systemic capacities to manage knowledge. Malawi has a national health research focal point and a R4H program housed within the Ministry of Health. The program has a health research mission statement, clearly defined terms of reference and an organizational structure. It has five technical and three support staff. Each technical staff in the program has a computer, an essential research tool. The program has e-mail and internet connectivity, so researchers can easily network and collaborate with peers within and outside the country, access pertinent data and literature from around the world and submit their manuscripts online for publishing.

Malawi has four public universities: the University of Malawi comprising the College of Medicine, the Kamuzu College of Nursing, Chancellor College, and the Polytechnic; Mzuzu University; Malawi University of Science and Technology; and Lilongwe University of Agriculture and Natural Resources. There are 11 private universities with varied capacities [33]. For example, the Catholic University of Malawi, the Malawi Adventist University and the University of Livingstonia were ranked 19 590, 21 117 and 21 276, respectively, among universities worldwide [34]. Whereas some colleges and research centres in the public universities have relatively strong capacity for R4H, that capacity in private universities is moribund. Nevertheless, all public and private universities constitute pillars upon which requisite R4H capacities could be built or strengthened. The faculties of health sciences in the public universities do not have memoranda of understanding with the Ministry of Health. Such memoranda could have been for developing human resources, providing technical advice, or undertaking research for the ministry. We concur with Nachega *et al.* [35] that African countries with limited levels of human resources for health research like Malawi ought to invest more in postgraduate training programs in epidemiology and public health. In addition, we share their view that African countries could accelerate building of a critical mass of epidemiologists through South-South and North-South collaboration.

Cognizant of the weakness in its resources for R4H, the Government of Malawi initiated the Health Research Capacity Strengthening Initiative with the support of the United Kingdom’s Department for International Development, Wellcome Trust and International Development Research Centre, with an overall goal of building and strengthening individual and institutional health research capacity. At the individual level, the initiative provides training fellowships, research grants, small grants, internships and PhD bursaries. For institutions, the initiative offers institutional grants and small grants for undergraduate dissertation work [36,37]. The initiative supported capacity strengthening at Chancellor College, the College of Medicine, the Polytechnic, Mzuzu University, Kamuzu College of Nursing, and Bunda College (now part of the Lilongwe University of Agriculture and Natural Resources). Program evaluation during August–October 2013 revealed that the initiative supported about 50 MSc and PhD students and over 400 undergraduate health-related projects in areas ranging from basic science to biomedical and social science [38]. Thus, the initiative has contributed to raising the number of scientists in Malawi and to promoting research interest among young Malawians. Malawi currently does not a have health research institute or council, and probably, as recommended by Mayosi and colleagues [39] for South Africa, new funding should be directed at developing such health research infrastructure.

The Ministry of Health plans to leverage the capacities developed by the Health Research Capacity Strengthening Initiative to build capacity for high quality health research at all levels of the national health system. That will be done through training of district health management teams and program staff in research methods applied to health systems and public health [2]. Furthermore, the ministry plans to ensure that monitoring, evaluation and epidemiology work, including surveillance, are strengthened and that the functionality of the health management information system is improved [2]. The objective is to provide reliable, complete, accessible, timely and consistent health-related information and ensure that it is used for evidence-based decision making at all levels of the health system.

### Producing and using R4H

The Malawi National Health Research Agenda 2012–2016 contains research priorities organized under nine disease and non-disease thematic areas: communicable diseases, noncommunicable diseases, sexual and reproductive health, trauma, mental health, nutrition, environmental health, health systems, and community system strengthening. The research priorities for each of the disease-based areas are categorized into epidemiology, prevention, diagnostics and treatment [2].

The health research program does not undertake research by itself but mainly identifies research needs and coordinates research work, for which it has a plan of action. Majority of R4H is conducted by the College of Medicine and Mzuzu University. Malawi does not have a national health research institute or council. According to the national health sector strategic plan 2011–2016, one of the persistent challenges is the limited multidisciplinary research, largely owing to the lack of highly qualified and experienced indigenous researchers [2]. However, that is improving. For example, between 2008 and 2013, aside from undergraduate projects, the Health Research Capacity Strengthening Initiative funded 15 doctor of philosophy, 41 Master of Science, 3 junior and 7 senior researchers, 1 multidisciplinary and 10 intern research projects. Out of the 77 projects, 49% were in biomedical science (clinical chemistry, microbiology, molecular, bio-statistics), 26% in public or international health (epidemiology, demography and informatics) and 25% in social science (anthropology and economics). By October 2013, 50% of the grantees had presented research results in conferences, 50% had submitted manuscripts for publication in various international journals and 6% had published papers in international journals [38].

During 2005–2012 the University of Malawi published a total of 443 articles in international journals [30]. About 215 (48.5%) of those were on a health subject, and of these 66% were by the College of Medicine, 10.7% by Kamuzu College of Nursing, 16.3% by Chancellor College, 3.7% by the Polytechnic and 3.3% by Bunda College of Agriculture. A limitation of the bibliometric study by Kakhongwe [40] was that it did not include articles published in national academic journals.

Uthman and Uthman’s [41] analysis of African countries’ biomedical papers indexed by PubMed between 1996 and 2005 showed that Malawi had a total of 450 articles, ranking 15 in contributions among the African countries indexed. Malawi had a relative growth of 67.6%.

Muula [42] quantified the publications from Kamuzu College of Nursing faculty since its opening in 1979 to mid-2006 indexed in Medline/PubMed, Psychinfo and Web of Science and found that 57 faculty members had contributed a total of 42 articles or there were 0.74 articles per faculty member. He attributed the low scholarly output to high teaching loads, lack of graduate study opportunities with 35% of staff having only an undergraduate degree, lack of research training, lack of competition from any other nursing school, lack of research funding, and dearth of role models.

Gondwe and Kavinya’s [43] search of the MEDLINE/PubMed database found 489 health articles originated from Malawi between 1996 and 2006. About 20.9% of these had Malawian first authors. There was a 103% increase in articles published in the 10 year period [43]. Clearly, those conducting R4H in Malawi have made commendable strides over the years, and they should be encouraged to intensify their efforts [44].

Malawi has a relatively new platform that brings together policy-makers, subject experts and researchers and that is designed for translating, synthesizing and communicating research to inform health policy and practice. That is expected to change the current landscape characterized by limited utilization of health research findings in practice and policy formulation owing to the limited interaction between researchers and users of the research findings [2]. For example, the University of Malawi and Mzuzu University, the two national universities with faculties of health sciences, did not have a memorandum of understanding with the Ministry of Health until recently. The main avenues for disseminating research findings have been national and institutional meetings, academic print media, conferences and scientific journals. However, very few policy-makers participate in these forums, and in many cases the dissemination process does not target or address the needs of non-technical policy-makers.

To promote the utilization of research findings for policy and program formulation, the health ministry plans to (a) create a website for the Research Unit and the National Health Sciences Research Committee; (b) establish a health policy analysis unit to produce policy briefs and newsletters; (c) require that national universities with faculties of health sciences such as the College of Medicine, Mzuzu University and Malawi College of Health Sciences implement the memorandum of understanding with the ministry; (d) develop leadership capacity for the integration of public health research into policy formulation and program planning; (e) organize annual conferences for dissemination of health research findings; and (f) promote evidence-based policy debates. The NCST is in the process of developing a registry of research to capture protocols and ethics submissions and to track fulfilment of the research agenda [38].

There will be need in future for an up-to-date bibliometric study that analyses in details Malawi’s health research performance over a period of one decade. Unlike the Kakhongwe study [40], the suggested study should include all articles published in national and international journals. Examples of such studies are those by Pouris [31], Senkubuge and Mayosi [21], Schneider [45] and the World Bank and Elsevier [46]. In addition, in order to monitor the alignment of research with the national R4H priority agenda, there is need for studies that compare the actual published research with national research priorities.

### Financing of R4H

In this paper financing refers to estimation of recurrent and capital cost of R4H; mobilization of funds for R4H from individuals, businesses including for-profit private firms and private non-profit organizations, government, bilateral and multilateral partners. and international foundations; accumulation and management of R4H funds; allocation of funds to individuals, institutions and networks within NHRS’s that govern and create R4H inputs and produce, monitor and evaluate R4H; and tracking expenditure on R4H.

In Malawi, R4H is primarily financed by the government and international nongovernmental organizations and to a lesser extent by multilateral and bilateral donors. Various mechanisms exist for funding health research, including institutional grants usually targeted at students and faculty in tertiary education institutions; national small grants that target the general research fraternity; and commissioned research grants from government departments, donors, nongovernmental organizations and public–private partnerships. The government plans to create a national health research fund to pool resources from the government and development partners for research in priority areas. The fund will be managed by the Ministry of Health.

The Ministry of Health has a budget line for research for health. Over the five-year financial period 2011/12–2015/16 Malawi budgeted 521 million Malawi kwacha (US$ 3.42 million) for improving the functioning of the health management information system to provide reliable, complete, accessible, timely and consistent health-related monitoring and evaluation information, and 139 million kwacha (US$ 0.913 million) for implementing the national health research agenda [2]. The budget document estimates that ideally to execute the activities planned under monitoring and evaluation type of research would require 782 million kwacha (US$ 5.15 million), and implementation of the research agenda would need 209 million kwacha (US$ 1.37 million). This is an acknowledgment that the R4H budget has a deficit of about 331 million kwacha (US$ 2.17 million).

The planned budget for research for the 2011/12–2015/16 financial years is approximately 0.26% of the total government budget of 252.154 billion kwacha (US$ 1.66 billion million) (Table 5) [2]. In the 2012/2013 financial year the Ministry of Health spent 28 million kwacha (US$ 0.18 million) on R4H (monitoring and evaluation was not included), which was 0.78% of the overall health budget of 3.6 billion kwacha (US$ 23.64 million) [47]. These figures do not include research and development expenditures by the commercial or private sector market, global health initiatives such as the Global Fund to Fight AIDS, Tuberculosis and Malaria, the GAVI Alliance and the Global Health Initiative or philanthropic contributions from private and public partners.

**Table 5:** **Malawi government’s R4H budget estimates for the HSSP period 2011–2016**

**Broad activities**	**Estimated budget (Malawi kwacha million)**						
	**2011/12**	**2012/13**	**2013/14**	**2014/15**	**2015/16**	**Total**	**Ideal cost 2011–16**
(A) M&E – development and research	88	94	103	108	127	521	782
(B) Implement national health research agenda	24	25	28	29	34	139	209
(C) Total (A+ B)	112	119	131	137	161	660	991
(D) Total for health budget	35 861	48 867	55 211	50 485	61 730	252 154	632 645
% = (C/D)*100	0.312	0.244	0.237	0.271	0.261	0.262	0.157

Source: Government of Malawi [2].

As Senkubuge and Mayosi [21] report for South Africa in their study on the state of NHRS in that country, Malawi also grossly under-invests in R4H. There is need, therefore, for the Ministry of Health to continue advocating to have 2% of the national health budget spent on research, in line with the recommendations of the Commission on Health Research for Development [48] and as endorsed by the ministers of health in Abuja [13], Accra [14], Algiers [12] and Bamako [11]. Advocacy is needed to have at least 5% of the health sector project and program aid from development aid agencies earmarked for R4H capacity strengthening as recommended by the Commission on Health Research for Development [48] and reiterated by the Fifty-eighth World Health Assembly [15].

We concur with Senkubuge and Mayosi [21] and Mayosi *et al.* [39] that to develop a robust NHRS every country requires a functional monitoring and evaluation mechanism created within existing R4H structures to serve a feedback-loop role. Such a mechanism is already being used in the Organization for Economic Co-operation and Development (OECD) countries [49], and, in fact, an annex in the OECD Frascati manual is considered relevant for developing countries and has been proposed for use in measuring their research and development programs [50]. Malawi needs to adapt the OECD mechanism to review its health needs, assess health-related research and development opportunities and status, and monitor expenditure on health-related research, development and innovation for financial resources from all sources, including the government, higher education, global health initiatives, philanthropic institutions and industry.

### Malawi NHRS index

**Figure 2:**
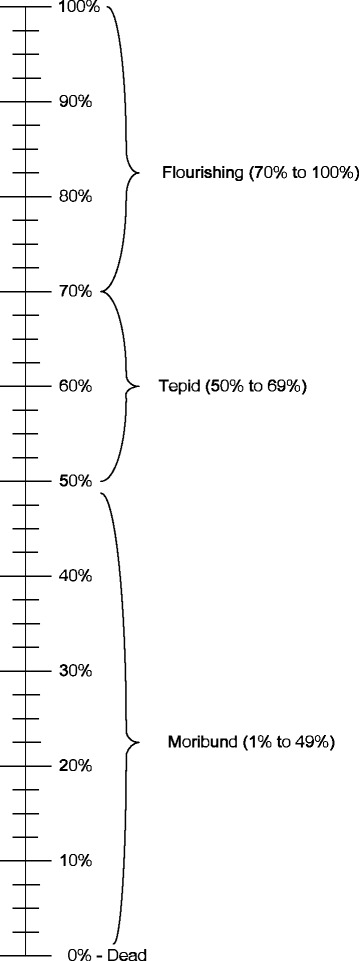
**Malawi national system of research for health gauge/scale.**

The Malawi R4H system of has four functions under which fall 30 sub-functions. Taking into account the information contained in the completed questionnaire on the Malawi health research program (see the “NHRS conceptual framework” section), we assessed the sub-functions and allocated them a percentage score ranging from 0%, if they were non-existent or dead, to 100%, if their performance was excellent or they were flourishing (see Figure 2). The Malawi national system of research for health index (MNSR4HI) that we constructed is the sum of the 30 sub-functions (SFI) indices listed in Table 6 divided by 30 and multiplied by 100%.

**Table 6:** **Malawi national system of research for health index (MNSR4HI)**

**Functions**	**Actual score (A)**	**Maximum score (B)**	**Minimum score (C)**	**Sub-function index (D) = (A-C)/(B-C)**
Governance of research for health				
National health policy	100	100	0	1
National health sector strategic plan	100	100	0	1
National policy on research for health	0	100	0	0
Strategic plan on research for health	0	100	0	0
Law governing research	95	100	0	0.95
National research for health priority list/agenda	98	100	0	0.975
National ethics review committee	90	100	0	0.9
Institutional ethical review committees	88	100	0	0.875
National R4H management forum	50	100	0	0.5
National scientific research committee	50	100	0	0.5
Developing and sustaining resources				
University colleges of health sciences conducting research	78	100	0	0.775
Availability of memorandum of understanding between university and ministry of health (MoH)	38	100	0	0.375
National health research institute(s) or council	0	100	0	0
Private universities conducting research for health	15	100	0	0.15
Health Management Information System doing regular monitoring and evaluation	50	100	0	0.5
Health research programme at MoH	80	100	0	0.8
National health research focal point	75	100	0	0.75
Public health laboratories	48	100	0	0.475
Libraries with access to latest journal issues	55	100	0	0.55
Producing and using research				
Existence of knowledge translation platform	40	100	0	0.4
Peer reviewed publications per person in population (compared to AFR average)	38	100	0	0.375
Use of research in development of new tools to improve health	25	100	0	0.25
Availability of computers in research programme	73	100	0	0.725
Availability of internet connectivity in research programme	83	100	0	0.825
Number of technical staff in a research for health programme	45	100	0	0.45
Financing				
Existence of a budget line in the health budget for research for health	60	100	0	0.6
Progress towards the target of allocating 2% of national health budget on R4H	16	100	0	0.155
Progress towards the target of allocating 5% of health-related project funding on research for health	8	100	0	0.075
Existence of NGOs funding research	0	100	0	0
Diversified research for health financial portfolio (public, industry, philanthropy)	35	100	0	0.35
Sum of sub-function indices (E)				15.3
Total Number of sub-functions (F)				30
MNSR4HI = [E/F)x100%				51

All the indices for the individual sub-functions were calculated using the following general formula:

$$ Sub\  function\  index=\left(\frac{Actual\  xi\  score - Minimum\  xi\  Score}{Maximum\  xi\  score- Minimum\  xi\  score}\right), $$

Where x_i_ is the i^th^ sub-function, such as the existence of a national policy on research for health (NPR4H), a strategic plan on research for health, a national research for health priority list/agenda, a national ethics review committee, institutional ethical review committees, a national R4H management forum, a law governing research, a national scientific research committee, etc. For instance, the national policy on research for health index (R4HPI) was calculated as follows:

$$ R4HPI=\left(\frac{Actual\ R4HP\kern0.5em - Minimum\ R4HP}{Maximum\ R4HP- Minimum\ R4HP}\right), $$

Where Actual R4HP is the actual research for health policy score, Minimum R4HP is the minimum research for health policy score, and Maximum R4HP is the maximum research for health policy score. As an example, if we assume that the regional minimum R4HP score is 0, the maximum score is 100 and the actual average R4HP score in Table 6 is 100, the R4HPI can be obtained as follows:

$$ R4HPI=\left(\frac{100\kern0.5em -0}{100-0}\right)=1. $$

Similarly, the national ethics review committee index (NERCI) was estimated as follows:

$$ NERCI=\left(\frac{Actual\  NERC\kern0.5em - Minimum\  NERC}{Maximum\  NERC- Minimum\  NERC}\right) $$

Where *Actual NERC* is the actual national ethics review committee (NERC) score (i.e. an average of the score from the Department of Research at the Ministry of Health and Dr Adamson Muula of the College of Medicine), *Minimum NERC* is the minimum NERC score, and *Maximum NERC* is the maximum NERC score. For example, if we assume that the regional minimum NERC score is 0, the maximum score is 100 and the actual average NERC score in Table 6 is 88, NERCI can be obtained as follows:

$$ NERCI=\left(\frac{88\kern0.5em -0}{100-0}\right)=0.88. $$

The indices for all the sub-functions were estimated in this way. Out of 30 sub-functions, 4 had an index of zero meaning dead or non-existent; 10 had 1 to 49% denoting moribund; 5 had 50 to 69% indicating tepid, and 12 had 70 to 100% denoting flourishing. The average indices were 67% for the function of governance of R4H; 48.6% for creating and sustaining R4H resources; 50.4% for producing and using R4H; and 23.6% for financing R4H.

Indices for individual sub-functions can aid the government to identify the reason for the sub-optimal performance of the R4H system or a component and to develop relevant interventions to improve specific sub-functions.

After appraising the individual sub-function indices, the overall MNSR4HI was calculated as follows:

$$ MNSR4HI=\left(\frac{{\displaystyle {\sum}_{i=1}^{30}}SFI}{T{N}_{SF}}\right)=\left(\frac{15.3}{30}\right) = 0.51\ x\ 100\%=51\% $$

Where SFI is the sub-function index, ${\displaystyle \sum_{i=1}^{30}}SFI$ is the summation from R4H sub-functions 1 to 30, and *TN*_*SF*_ is the total number of R4H sub-functions, which is equal to 30 in this study.

Since the national R4H index is measured on a scale of 0 (or 0%) to 1 (or 100%), the MNSR4HI of 0.51 (or 51%) implies that Malawi’s R4H performance is average.

The formula we developed for the national R4H index is very similar to that used by the United Nations Development Programme to calculate the human development index [51]. It is also comparable to that used by Kirigia and Kirigia [52] in developing a Health Development Governance Index. In this paper we categorized national systems of R4H with an overall index of 0% as dead or non-existent, those with a score of 1% to 49% as moribund, those with a score of 50% to 69% to be tepid, and those with a score of 70% to 100% to be flourishing. Since the *MNSR*4*HI* is within the 50% to 69% bracket, the Malawi national system of research for health is judged to be tepid. Of course, we consider this index as a rough indicator of the status of the Malawi national research for health system and as first step in its assessment. We expect other researchers to treat our study as a work-in-progress for debate and refinement so that a generally agreed-upon index can emerge for wide application in the African Region or globally. We also believe that even the indices for Malawi could be refined further if they were drawn up by a wider sample of R4H researchers. It is our hope that this index process will trigger heated debate globally.

## Summary

A functional NHRS is a prerequisite for the achievement of health system goals of improving health and health equity in ways that are responsive, financially fair, and make the most efficient use of available resources [53]. Volmink and Dare [54] characterized African research – and by implication national health research systems – as moribund. In our view, however, the Malawi government, with partner support, has made substantive effort to strengthen capacities of individuals and institutions that generate scientific knowledge, and so we consider its NHRS as tepid with significant potential to flourish. As shown in Figure 2, the *MNSR*4*HI* is 51%, which is within the 50%-69% range.

### RH4 governance

Governance of R4H has improved with the promulgation of the Malawi Science and Technology Act in 2003; the establishment of the NCST to regulate the conduct of research and the National Health Sciences Research Committee and COMREC to ensure technical and ethical rigor of research protocols and proposals; and development of a national health research agenda. However, lack of an explicit R4H policy, a strategic plan or a national health research management forum greatly undermines the government’s effectiveness in supervising the NHRS.

### Developing and sustaining R4H resources

Malawi has a national health research focal point and a R4H program housed within the Ministry of Health. There are four public and 11 private universities, which could be pillars upon which requisite R4H capacities could be built. Efforts are under way in the Ministry of Health to strengthen the functionality of the health management information system to facilitate the conduct of implementation research. In our view, the Malawi government with partner support has made substantive effort to strengthen capacities of individuals and institutions that generate scientific knowledge. However, as acknowledged in various government documents and articles published by Malawian researchers, much more remains to be done to strengthen the capacities of both public and private institutions of higher learning to increase R4H publications.

### Producing and using R4H

The health research program does not undertake research by itself. During the period 2005–2012 the University of Malawi published 215 articles on various aspects of public health. Uthman and Uthman’s [41] analysis of African countries’ biomedical papers indexed by PubMed between 1996 and 2005 revealed that Malawi had a total of 450 articles, which earned it a rank of 15 among African countries. Gondwe and Kavinya’s [43] search of the MEDLINE/PubMed database found 489 articles on health originating from Malawi. A Malawian scholar [44] has argued that while there has been growth in the number of publications, efforts need to be intensified to boost research productivity in the country. So far the utilization of research findings in policy development and public practice can best be described as tepid. However, use of knowledge from research in decision making is expected to improve in the medium and long term with the creation of the knowledge translation platform.

### Financing

R4H is primarily financed by the Government of Malawi and international nongovernmental organizations and to a lesser extent by multilateral and bilateral donors. The Ministry of Health has a budget line for research for health. The budget planned for research for the five financial years 2011/12–2015/16 is approximately 0.26% of the total government budget of 252 154 million kwacha [2]. The level of funding for R4H is far lower than 2% of the national health budget, the level recommended by the Commission on Health Research for Development [48].

### Ethical clearance

The study was exempted from review by the Malawi COMREC based on the understanding that it would be based solely on existing public data, documents and records and completion of the questionnaire by the two Malawian co-authors.

## Abbreviations

CHAM: Church Association of Malawi
COMREC: College of Medicine Research and Ethics Committee
Int$: International dollars or purchasing power parity
MNSR4HI: Malawi national system of research for health index
NCST: National Commission for Science and Technology
NHRI: National Health Research Institute
R4H: Research for health
SFI: Sub-function index
WHO: World Health Organization
